# Increased carbapenemase testing following implementation of national VA guidelines for carbapenem-resistant Enterobacterales (CRE)

**DOI:** 10.1017/ash.2021.220

**Published:** 2022-06-02

**Authors:** Margaret A. Fitzpatrick, Katie J. Suda, Swetha Ramanathan, Geneva Wilson, Linda Poggensee, Martin Evans, Makoto M. Jones, Christopher D. Pfeiffer, J. Stacey Klutts, Eli Perencevich, Michael Rubin, Charlesnika T. Evans, for the QUERI CARRIAGE Program

**Affiliations:** 1 Department of Veterans’ Affairs, Center of Innovation for Complex Chronic Healthcare, Edward Hines, Jr, VA Hospital, Hines, Illinois; 2 Division of Infectious Diseases, Department of Medicine, Loyola University Chicago Stritch School of Medicine, Maywood, Illinois; 3 Department of Veterans’ Affairs, Center of Health Equity Research & Promotion, VA Pittsburgh Healthcare System, Pittsburgh, Pennsylvania; 4 Department of Medicine, University of Pittsburgh School of Medicine, Pittsburgh, Pennsylvania; 5 Department of Veterans’ Affairs, Lexington VA Medical Center, Lexington, Kentucky; 6 Department of Veterans’ Affairs, VA Salt Lake City Healthcare System, Salt Lake City, Utah; 7 Division of Epidemiology, Department of Medicine, University of Utah, Salt Lake City, Utah; 8 Department of Veterans’ Affairs, Portland VA Healthcare System, Portland, Oregon; 9 Division of Infectious Diseases, Department of Medicine, Oregon Health Science University, Portland, Oregon; 10 Center for Access & Delivery Research and Evaluation, Department of Veterans’ Affairs, Iowa City VA Health Care System, Iowa City, Iowa; 11 Department of Pathology, University of Iowa Carver College of Medicine, Iowa City, Iowa; 12 Department of Internal Medicine, University of Iowa Carver College of Medicine, Iowa City, Iowa; 13 Center for Health Services and Outcomes Research and Department of Preventive Medicine, Institute for Public Health and Medicine, Northwestern University Feinberg School of Medicine, Chicago, Illinois

## Abstract

**Objective:**

To describe national trends in testing and detection of carbapenemases produced by carbapenem-resistant Enterobacterales (CRE) and associate testing with culture and facility characteristics.

**Design:**

Retrospective cohort study.

**Setting:**

Department of Veterans’ Affairs medical centers (VAMCs).

**Participants:**

Patients seen at VAMCs between 2013 and 2018 with cultures positive for CRE, defined by national VA guidelines.

**Interventions:**

Microbiology and clinical data were extracted from national VA data sets. Carbapenemase testing was summarized using descriptive statistics. Characteristics associated with carbapenemase testing were assessed with bivariate analyses.

**Results:**

Of 5,778 standard cultures that grew CRE, 1,905 (33.0%) had evidence of molecular or phenotypic carbapenemase testing and 1,603 (84.1%) of these had carbapenemases detected. Among these cultures confirmed as carbapenemase-producing CRE, 1,053 (65.7%) had molecular testing for ≥1 gene. Almost all testing included KPC (n = 1,047, 99.4%), with KPC detected in 914 of 1,047 (87.3%) cultures. Testing and detection of other enzymes was less frequent. Carbapenemase testing increased over the study period from 23.5% of CRE cultures in 2013 to 58.9% in 2018. The South US Census region (38.6%) and the Northeast (37.2%) region had the highest proportion of CRE cultures with carbapenemase testing. High complexity (vs low) and urban (vs rural) facilities were significantly associated with carbapenemase testing (*P* < .0001).

**Conclusions:**

Between 2013 and 2018, carbapenemase testing and detection increased in the VA, largely reflecting increased testing and detection of KPC. Surveillance of other carbapenemases is important due to global spread and increasing antibiotic resistance. Efforts supporting the expansion of carbapenemase testing to low-complexity, rural healthcare facilities and standardization of reporting of carbapenemase testing are needed.

Carbapenem-resistant Enterobacterales (CRE) are difficult-to-treat multidrug-resistant organisms (MDROs) associated with high morbidity and mortality and the potential for rapid spread.^1–3^ CRE are 1 of the US Centers for Disease Control and Prevention (CDC) 5 most urgent antimicrobial-resistant threats.^4^ Recent national epidemiologic surveillance data have demonstrated decreased incidence of most MDROs, but no change in both hospital and community-onset CRE incidence between 2012 and 2017.^5^ Prompt laboratory identification of CRE and delineation of whether CRE produces a carbapenemase (‘carbapenemase-producing CRE’ or ‘CP-CRE’) are critical to controlling spread through rapid implementation of infection control measures and earlier initiation of appropriate antimicrobial treatment. Furthermore, categorization of the type of carbapenemase enzyme produced by CRE is important because different enzymes are associated with unique geographic distributions, epidemiologic risks, and antibiotic susceptibilities.^6,7^ All 4 major carbapenemase enzymes have been identified from CRE in the United States: *Klebsiella pneumoniae* carbapenemase (KPC), New Dehli metallo-β-lactamase (NDM), verona-integron–encoded metallo-β-lactamase (VIM), imipenemase (IMP), and oxacillinase-48–like (OXA-48) enzymes. KPC remains the most common enzyme detected, present in almost 90% of all CRE isolates submitted for testing to the CDC Antibiotic Resistance Laboratory Network in 2017 and 2018.^8^

Laboratory practices for identification and characterization of CP-CRE have rapidly changed, with newer molecular techniques, such as PCR, making identification faster and more sensitive compared to older phenotypic tests such as the modified Hodge test (MHT).^9^ The Veterans’ Health Administration within the Departent of Veterans’ Affairs (VA) has been a national leader in developing guidelines for the detection, management, and prevention of CRE and CP-CRE. The VA first released national CRE guidelines in 2015, which included an algorithm for laboratory detection of CRE based on antibiotic susceptibility criteria and guidelines for performing MHT in certain circumstances.^10^ The VA issued new 2017 guidelines (released on December 15, 2016) prioritizing CP-CRE identification by simplifying antibiotic susceptibility criteria and recommending PCR to confirm carbapenemase production.^11^ Since the release of the 2017 guidelines, most VA laboratories have followed the updated guidelines for initial CRE identification using antibiotic susceptibility criteria, but only half use PCR to confirm carbapenemase production.^12^ Studies describing the VA experience with CRE guideline implementation can serve as key sources of data and support for private sector hospitals developing similar programs.

The overall goal of this study was to analyze trends in carbapenemase testing and detection in VAMCs following the dissemination of VA CRE guidelines. We also identified culture and facility-level characteristics associated with carbapenemase testing. Finally, we aimed to describe specific testing for and detection of both KPC and non-KPC (VIM, IMP, NDM, OXA-48) enzymes.

## Methods

### Study setting and design

This retrospective cohort study included adult patients with CRE at all VA medical centers (VAMCs) between January 1, 2013, and December 31, 2018. Data were extracted from the VA Corporate Data Warehouse (CDW), a national repository of clinical and administrative data from Veterans’ Health Administration (VHA) electronic medical records updated on a continual basis. CDW data were used to obtain microbiology and laboratory data and care setting: outpatient setting (including clinic or emergency department), inpatient setting, or long-term care.

### CRE definitions and carbapenemase testing

The VA definition for CRE and CP-CRE changed over the study period; thus, we included patients with cultures that met either or both definitions. The first definition from the 2015 guidelines included *Escherichia coli*, *Klebsiella* spp, and *Enterobacter* spp that were (1) nonsusceptible to imipenem, meropenem, and/or doripenem or were resistant to ertapenem and (2) resistant to any tested third-generation cephalosporin.^10^ The guidelines emphasized current Clinical and Laboratory Standards Institute (CLSI) carbapenem break points (M100-S21 or higher at that time) but also provided algorithmic guidance for laboratories using earlier CLSI break points. These guidelines also recommended confirmation of carbapenemase production using MHT. The second CRE definition from the 2017 guidelines included *Escherichia coli*, *Klebsiella pneumoniae*, *K. oxytoca*, and *Enterobacter* spp and simplified the antibiotic susceptibility criteria to resistance to imipenem, meropenem, and/or doripenem.^11^ The 2017 guidelines required laboratories to use CLSI M100-S21 or higher. In the interim between the 2015 and 2017 guidelines, the Food and Drug Administration (FDA) approved a molecular platform to identify carbapenemase genes^13^; therefore, the 2017 guidelines also required PCR to identify carbapenemase genes, with the recommendation to use the FDA-approved platform.

Patients with standard cultures from any site that grew *Escherichia coli*, *Klebsiella* spp, and/or *Enterobacter* spp and met either or both the 2015 and 2017 VA CRE definitions were included. The main analysis did not exclude subsequent cultures; therefore, >1 culture per patient could be included. A subgroup analysis was performed including only the first CRE culture per patient to determine whether differences in testing on subsequent cultures may have affected our results. Cultures with ‘rectal’ labeled as the site were included in the main cohort if they had full microbiology identification and susceptibility testing performed and met a VA CRE definition. Direct PCR tests for carbapenemase genes performed from rectal or stool specimens without associated microbiologic cultures were not included. A subgroup analysis was also performed excluding rectal cultures. Bacterial species identification and antibiotic susceptibility testing was performed by each VAMC laboratory per their own protocols.

Types of carbapenemase tests were initially extracted from CDW microbiology and laboratory reports by identifying the names of phenotypic tests included in the report: carbapenem inactivation method (CIM), MHT, Rapidec Carba NP (Biomerieux, Durham, NC), matrix associated laser desorption ionization-time of flight (MALDI-TOF)] and/or genotypic tests such as polymerase chain reaction (PCR) testing including Xpert Carba R (Cepheid, Sunnyvale, CA). For the remainder of this manuscript, the term ‘carbapenemase testing’ will encompass all types of tests, unless specifically delineated. In addition, unstructured data fields in reports were reviewed for text strings that indicated carbapenemase testing but did not specify a name (eg, ‘carbapenemase positive’). Test names and text strings were reviewed manually by an infectious diseases (ID) physician and ID pharmacist; those records that did not indicate a carbapenemase test after manual review were removed. The remaining data were categorized by which carbapenemase test was performed and whether results were positive or negative. The ID physician and ID pharmacist performed their reviews independently and then reconciled differences. For cultures with any carbapenemase test identified, data were also collected in a similar manner from text in microbiology reports on testing and detection of specific types of carbapenemase enzymes or genes (ie, KPC, NDM, VIM, IMP, and OXA-48).

### Facility characteristics

The CDW data were used to collect various characteristics of VAMC facilities where CRE cultures were identified. VAMCs are classified into 3 complexity levels determined in part by patient volume, patient characteristics, and research and teaching activities (levels 1a–c, 2, and 3, with level 1a being highest).^14^ We defined high-complexity facilities as levels 1a–c and low-complexity facilities as levels 2 and 3. The VA also uses the Rural–Urban Commuting Areas system to classify VAMCs into urban versus rural.^15^ Urban VAMCs are located in census tracts with at least 30% of the population residing in an urbanized area as defined by the US Census Bureau. Rural VAMCs are located in areas not defined as urban.

### Statistical analysis

Descriptive statistics summarized culture sources, care settings, bacterial species, and carbapenemase testing for unique CRE cultures and facility characteristics for unique VAMCs where CRE cultures were obtained. Bivariate statistics determined using the χ^2^ or Fisher exact test were used to associate culture- and facility-level variables with carbapenemase testing overall, as well as testing for non-KPC genes or enzymes. *P* < .05 was considered significant. Statistical analyses were conducted out using SAS version 9.4 software (SAS Institute, Cary, NC) and Stata version 12.1 software (StataCorp, College Station, TX).

## Results

### Overall carbapenemase testing and detection

**Fig. 1.**
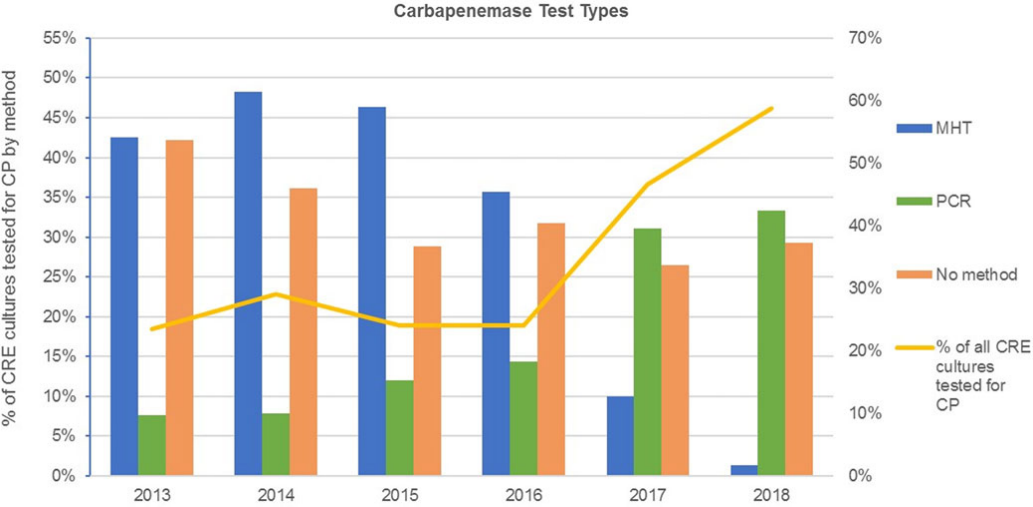
Trends over time in types of carbapenemase tests identified among carbapenem-resistant Enterobacterales (CRE) cultures. The orange line indicates the percentage of CRE cultures that had evidence of any test for carbapenemase production (CP), and the colored bars reflect the percentage of CRE cultures tested for carbapenemases with the indicated method (ie, MHT, modified Hodge test or PCR, polymerase chain reaction). Other methods reported in low frequency included carba-NP, carbapenem inactivation method, and matrix-assisted laser desorption ionization-time of flight (all <5%). The green arrow indicates the publication of updated VA guidelines (in early 2017) requiring PCR testing for carbapenemases among suspected CP-CRE isolates.

The 5,778 standard cultures that grew CRE were identified from 3,096 patients cared for at 132 VA facilities during the study period. Most CRE cultures were *Klebsiella* spp. Inpatients contributed the greatest proportion of cultures, but many (39.5%) CRE cultures were obtained from outpatients (Table 1). Overall, 1,905 CRE cultures (33.0%) had evidence of carbapenemase testing, with MHT being most commonly performed (Table 1 and Fig. 1). Moreover, 1,603 CRE cultures (84.1%) with evidence of carbapenemase testing had detection of carbapenemase enzymes and/or genes (ie, they were CP-CRE). For 95 of the 1,603 CP-CRE cultures (5.9%), there was no evidence of carbapenemase testing for that culture but the microbiology report referred to carbapenemase detection in a recent previous culture growing the same species; therefore, these cultures were also considered to be CP-CRE.

**Table 1.** Culture Characteristics Associated With Carbapenemase Testing

Culture Characteristic	CRE Cultures Tested for CP (n=1,905, 33.0%), No. (%)	CRE Cultures Not Tested for CP (n=3,873, 67.0%), No. (%)	*P* Value
**Organism**			
*Escherichia coli*	147 (24.9)	444 (75.1)	<.0001
*Klebsiella* spp	1,372 (34.9)	2,560 (65.1)	
*Enterobacte*r spp	386 (30.8)	869 (69.2)	
**Care setting at time of CRE culture**			
Inpatient	935 (34.4)	1,784 (64.6)	<0.0001
Outpatient	660 (28.9)	1,623 (71.1)	
Long-term care	310 (39.9)	466 (60.1)	
**Source**			
Blood	189 (39.3)	292 (60.7)	.0089
Urine	1163 (32.5)	2,410 (67.5)	
Respiratory	235 (30.5)	536 (69.5)	
Rectal	28 (41.8)	39 (58.2)	
Other	290 (32.7)	596 (67.3)	
**Year**			
2013	248 (23.5)	808 (76.5)	<.0001
2014	280 (29.0)	686 (71.0)	
2015	248 (24.1)	781 (75.9)	
2016	258 (24.1)	811 (75.9)	
2017	419 (46.7)	479 (53.3)	
2018	447 (58.8)	313 (41.2)	
**Type of carbapenemase test**			
CIM	3 (0.2)	…	
MHT	498 (26.1)	…	
Carba-NP	12 (0.63)	…	
MALDI-TOF	16 (0.84)	…	
PCR based	387 (20.3)	…	
Unknown or undetermined	989 (52)	…	

Note. CRE, carbapenem-resistant Enterobacterales; CP, carbapenemase production; CIM, carbapenem inactivation method; MHT, modified hodge test; MALDI-TOF, matrix-assisted laser desorption ionization time of flight; PCR, polymerase chain reaction.

Carbapenemase testing was relatively stable between 2013 and 2016, with ∼25%–30% of CRE cultures tested. Thereafter, testing significantly increased, with 46.7% and 58.8% of CRE cultures tested in 2017 and 2018, respectively. Interesting trends in test method were observed, with decreased frequency of MHT and increased frequency of PCR testing over the study period (Fig. 1). Similarly, the proportion of CRE cultures with a carbapenemase test performed but for which the specific method could not be identified decreased from 42.2% in 2013 to 29.3% in 2018 (Fig. 1). Carbapenemase detection among tested isolates increased during the study period, with a high of 46.3% in 2018 compared with a low of 18.2% in 2016. Carbapenemase testing was more likely to occur for cultures that grew *Klebsiella* spp that were obtained in hospital or LTC settings and were from blood or rectal specimens (Table 1).

We performed a subgroup analysis including just the first CRE culture per patient. Among the 3,096 patients with CRE in the cohort, 1,088 (35.1%) contributed >1 CRE culture, and these cultures were excluded from the subgroup analysis. In this subgroup analysis, the proportion of CRE cultures tested for carbapenemases, the increase in carbapenemase testing over time, and the culture characteristics associated with carbapenemase testing did not significantly differ from the main analysis (Supplementary Table 1). We detected a slightly lower frequency of cultures with undetermined type of carbapenemase test performed: 52% for all CRE culture cohort versus 42.1% for first culture per patient cohort. Similarly, after excluding the 67 rectal CRE cultures (1.2%), we did not detect any significant differences in these results compared to the main analysis.

### Testing and detection of specific carbapenemase enzymes

**Fig. 2.**
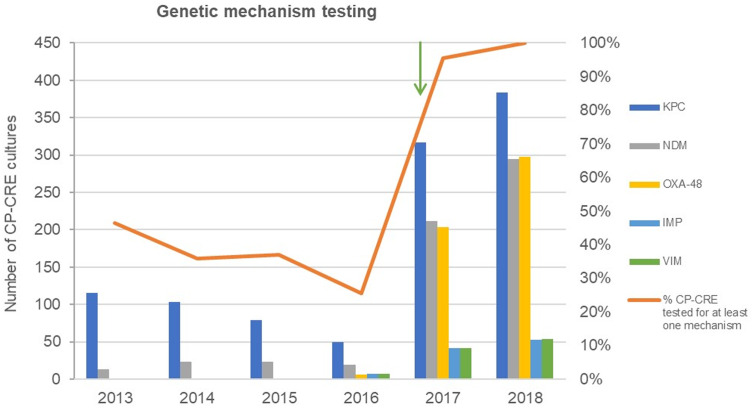
Trends over time in testing for carbapenemase genes among cultures that grew carbapenemase-producing carbapenem-resistant Enterobacterales (CP-CRE). The orange line indicates the percentage of CP-CRE cultures that were subsequently tested for at least 1 genetic mechanism of resistance, and the colored bars reflect the number of CP-CRE cultures with evidence of testing for each specific gene. The green arrow indicates the publication of updated VA guidelines (in early 2017) requiring PCR testing for carbapenemases among suspected CP-CRE isolates.

Of the 1,905 CRE cultures tested for carbapenemases, 1,053 (55.3%) also had evidence of testing for at least 1 specific mechanism of carbapenemase production. Of these 1,053 cultures, 1,047 (99.4%) were tested for KPC. NDM was the second most common mechanism (n = 585, 55.6%), followed by OXA-48 (n = 507, 48.1%), VIM (n = 102, 9.7%), and IMP (n = 102, 9.7%). Similar to carbapenemase testing overall, specific tests for KPC, NDM, and OXA-48 enzymes increased substantially in 2017 and 2018 (Fig. 2). Increases were also observed in tests for VIM and IMP enzymes in 2017 and 2018, but to a lesser extent (Fig. 2). KPC was detected in 914 (87.3%) of 1,047 cultures, whereas NDM (n = 8 of 585, 1.4%) and OXA-48 (n = 1 of 507, 0.2%) were rarely detected. No CRE cultures had VIM or IMP enzymes. The absolute number of CRE cultures with KPC detected increased over the study period (108 in 2013 vs 336 in 2018), although the detection rate (ie, the number of CRE cultures with KPC detected divided by the number of CRE cultures with KPC tested) decreased slightly from 93.9% in 2013 to 83.5% in 2018.

### Association of carbapenemase testing with facility characteristics

Table 2 displays the associations between various facility characteristics and carbapenemase testing. Overall, most CRE cultures were from high complexity VAMCs located in urban areas. The greatest proportions of CRE cultures tested for carbapenemases were seen in the Northeast US Census region [432 (37.2%) of 1,160] and the South region [659 (34.7%) of 1,708; *P* < .0001]. Furthermore, a significantly greater proportion of CRE cultures were tested for carbapenemases in urban VAMCs (*P* = .03), affiliated with academic medical centers (*P* = .01), and with high complexity level (*P* < .0001). The facility characteristics associated with carbapenemase testing in the subgroup analysis including just the first CRE culture per patient remained largely unchanged compared to the main analysis (Supplementary Table 2).

**Table 2.** Facility Characteristics Associated With Carbapenemase Testing for CRE Cultures

Facility Characteristic	CRE Cultures Tested for CP (n=1,905, 33.0%), No. (%)	CRE Cultures Not Tested for CP (n=3,873, 67.0%), No. (%)	*P* Value
**US Census region**			
Northeast	432 (37.2)	728 (62.8)	<.0001
Midwest	207 (30.1)	481 (69.9)	
West	213 (34.3)	408 (65.7)	
South	659 (34.7)	1,049 (61.4)	
Outside continental United States	394 (24.6)	1,207 (75.4)	
**Rurality**			
Rural	74 (26.9)	201 (73.1)	.03
Urban	1,831 (33.3)	3,672 (66.7)	
**AMC affiliation**			
Yes	1,884 (33.0)	3,814 (66.9)	.01
No	20 (25.0)	60 (75.0)	
**Complexity level**			
High	1,808 (33.6)	3,579 (66.4)	<.0001
Low	92 (23.5)	299 (76.5)	
**Transplant programs**			
None	1,695 (33.9)	3,302 (66.1)	<.0001
1–2 in-house programs or 3 sharing programs	173 (24.5)	534 (75.5)	
3+ in-house programs	37 (50.0)	37 (50.0)	

Note. CRE, carbapenem-resistant Enterobacterales; CP, carbapenemase production; AMC, academic medical center.

Many laboratories may focus on detection of KPC only because it is the most common carbapenemase enzyme detected in the United States.^8^ Therefore, we further examined facility characteristics associated with testing only for KPC versus testing for KPC and other carbapenemases. Of the 1,053 CP-CRE cultures for which there was evidence of at least 1 specific mechanism in the microbiology report, 457 cultures (43.4%) only had evidence of testing for KPC while 596 (56.6%) had evidence of testing for 1 or more non-KPC mechanisms. Nearly all mechanism testing was performed on CP-CRE cultures obtained from VAMCs associated with an academic medical center (n = 1050, 99.7%). Testing for any or all non-KPC mechanisms was more likely to occur outside of the continental United States, in high-complexity VAMCs, and in urban VAMCs (*P* < .0001 for all); however, we detected no significant difference by academic affiliation (*p* = .4155).

## Discussion

Identification of the type of carbapenemase produced by CP-CRE provides critical information for clinical care and empiric antibiotic treatment, helps guide real-time infection control response, and informs epidemiologic surveillance. National guidelines have recently emphasized the importance of PCR testing for specific carbapenemase genes in the overall laboratory management of CRE.^4,11^ Our study showed that between 2013 and 2018, 33% of standard cultures that grew CRE at VAMCs were tested for carbapenemases and >50% of these were tested for at least 1 specific genetic mechanism. Both overall carbapenemase testing and testing for specific mechanisms increased, with the greatest increases following dissemination of updated VA CRE guidelines in early 2017. Furthermore, use of less sensitive phenotypic tests, such as MHT, decreased over the study period, whereas the use of newer, more sensitive PCR-based tests increased. This result may partially explain the increase in carbapenemase detection in 2018 compared with earlier study years. Because a substantial proportion of CRE cultures with evidence of carbapenemase testing did not have data in the microbiology reports on the type of test performed, these trends should be interpreted with caution. However, these data are consistent with prior work showing that almost all VAMC laboratories used the 2017 VA CRE guidelines,^12^ which suggests that some laboratories rapidly incorporated the preferred molecular testing into their CRE algorithms as part of guideline implementation.^13^

Testing for specific genetic mechanisms also increased in 2017 and 2018. Among facilities that tested CP-CRE isolates for specific mechanisms, nearly all tested for KPC; however, increases were also observed in testing for other genes. PCR platforms allow for testing mutlple genetic mechanisms at once^6^; thus, the 2017 guideline update as well as increased PCR availability likely facilitated testing and identification of non-KPC genes. Identification of non-KPC carbapenemases is important for monitoring local and national trends in CP-CRE spread, particularly since these enzymes are more prevalent in other countries.^6^ Even with increased testing in 2017 and 2018, few non-KPC carbapenemases were detected, which is consistent with national CDC epidemiologic surveillance^8^ and prior VA data.^16^ An important caveat to this finding is that our CDW data extraction process could only assess cultures as being tested for specific mechanisms based on text in microbiology reports. Underreporting of tests for the less prevalent non-KPC mechanisms could have biased our results.

Carbapenemase testing was more likely to occur for cultures obtained in hospital or LTC settings, and from blood. Because the prevalence of CP-CRE is greater in hospitals and LTCFs compared to the community,^17^ laboratories may be prioritizing resources for carbapenemase testing to care settings at highest risk for CP-CRE and to the patients with more severe infections. Furthermore, high-complexity VAMCs located in urban areas and affiliated with academic centers were more likely to perform carbapenemase testing and to test for non-KPC carbapenemases. These facilities are more likely to care for medically complex patients with multiple CRE risk factors. The results of this study are consistent with prior work showing that laboratories in high-complexity, urban VAMCs were more likely than those in low-complexity, rural facilities to perform any carbapenemase testing and/or to use PCR specifically.^12^

An important limitation of this study was the inability to distinguish between patients infected versus colonized with CRE. However, our focus was on carbapenemase testing and associated facility-level characteristics rather than patient-level clinical care, and a subgroup analysis excluding rectal cultures did not show different results from the main analysis. Furthermore, although all VA laboratories are recommended to follow the lastest CLSI recommendations for carbapenem break points in Enterobacterales, we did not have access to which specific CLSI break points VA microbiology laboratories used. Therefore, the identification and reporting of bacterial susceptibilities may have changed during our study period, leading us to exclude cultures from earlier study years that may have been CRE if updated CLSBI break points had been used to report susceptibilities. As indicated above, individual laboratories may not have entered text into the culture report on all carbapenemase testing performed, particularly if tests were negative or if facilities sent CRE isolates to a reference laboratory. This factor may have led us to underestimate carbapenemase testing, although it is reassuring that the proportion of CRE cultures with evidence of a carbapenemase test but for which the method could not be identified decreased over the study period.

In conclusion, encouraging increases were observed in carbapenemase testing following publication of national VA CRE guidelines. However, as of 2018, >40% of cultures that grew CRE in all VAMCs and >75% of cultures in low-complexity, rural facilities did not have evidence of carbapenemase testing. Our study indicates a need to expand carbapenemase testing, to standardize test reporting in microbiology reports, and to support all laboratories in fully implementing national recommendations. In 2019, VA released an updated CRE tool kit introducing newer approaches to laboratory testing and infection prevention and promoting use of the CDC Antibiotic Resistance Laboratory Network (ARLN) and the VHA Inpatient Pathogen Tracker, among other changes.^18^ The findings from this study will need to be explored further to assess the impact of the 2019 tool kit on carbapenase testing and detection. Further research in this area could help delinate the most cost-effective strategies to enhance implementation of carbapenemase testing for both VA and private-sector healthcare systems.

## Supplementary material

For supplementary material accompanying this paper visit https://doi.org/10.1017/ash.2021.220. click here to view supplementary material
