# Supplementary material for: Increased carbapenemase testing following implementation of national VA guidelines for carbapenem-resistant Enterobacterales (CRE)
Source: Antimicrob Steward Healthc Epidemiol. 2022 Jun 2;2(1):e88. doi: 10.1017/ash.2021.220 (PMC9726513; doi:10.1017/ash.2021.220)
Supplement: Supplementary file 1 [file S2732494X21002205sup001.docx]

**Supplemental Table 1.** Culture characteristics associated with carbapenemase testing in the sub-group analysis including just the first CRE culture per patient.

| **Culture characteristic variable** | **CRE cultures tested for CP**  **n=989 (31.9%)** | **CRE cultures not tested for CP**  **n=2,107 (68.1%)** | **p-value** |
| --- | --- | --- | --- |
| Organism |  |  |  |
| *E.coli* | 99 (21.2) | 369 (78.9) | <0.0001 |
| *Klebsiella* spp. | 605 (34.8) | 1,132 (65.2) |  |
| *Enterobacte*r spp. | 285 (32.0) | 606 (68.0) |  |
| Care setting at time of CRE culture |  |  |  |
| Inpatient | 487 (34.9) | 907 (65.1) | <0.0001 |
| Outpatient | 393 (27.7) | 1,026 (72.3) |  |
| Long-term care | 109 (40.8) | 174 (61.2) |  |
| Source |  |  |  |
| Blood | 63 (40.4) | 93 (59.6) | 0.006 |
| Urine | 619 (30.6) | 1,403 (69.4) |  |
| Respiratory | 112 (30.2) | 259 (69.8) |  |
| Other | 179 (34.7) | 337 (65.3) |  |
| Year |  |  |  |
| 2013 | 125 (22.5) | 431 (77.5) | <0.0001 |
| 2014 | 129 (27.8) | 335 (72.2) |  |
| 2015 | 141 (26.1) | 399 (73.9) |  |
| 2016 | 144 (25.3) | 425 (74.7) |  |
| 2017 | 221 (40.9) | 320 (59.2) |  |
| 2018 | 229 (53.7) | 197 (46.3) |  |
| Type of carbapenemase test |  | -- |  |
| CIM | 3 (0.3%) | -- |  |
| MHT | 300 (30.3%) | -- |  |
| Carba-NP | 10 (1.0%) | -- |  |
| MALDI-TOF | 15 (%) | -- |  |
| PCR based | 245 (24.8%) | -- |  |
| Unknown or undetermined | 416 (42.1%) | -- |  |

CP, carbapenemase; CIM, carbapenem inactivation method; MHT, modified Hodge test; PCR, polymerase chain reaction

**Supplemental Table 2**. Facility characteristics associated with carbapenemase testing for CRE cultures in the sub-group analysis including just the first CRE culture per patient.

| **Facility characteristic variable** | **Value** | **CRE cultures tested for CP n=989 (31.9%)** | **CRE cultures not tested for CP**  **n=2,107 (68.1%)** | **p-value** |
| --- | --- | --- | --- | --- |
| Geographic region | Northeast | 170 (32.4) | 354 (67.6) | <0.0001 |
|  | Midwest | 140 (30.3) | 322 (69.7) |  |
|  | West | 130 (38.0) | 280 (68.3) |  |
|  | South | 393 (38.0) | 647 (62.0) |  |
|  | Outside continental U.S. | 156 (23.4) | 510 (76.6) |  |
| Rurality | Rural | 53 (26.0) | 151 (74.0) | 0.06 |
|  | Urban | 936 (32.4) | 1,956 (67.6) |  |
| AMC affiliation | Yes | 977 (32.2) | 2,059 (67.8) | 0.05 |
|  | No | 12 (20.0) | 48 (80.0) |  |
| Complexity level | High | 937 (33.2) | 1,884 (66.8) | <0.0001 |
|  | Low | 52 (18.9) | 223 (81.1) |  |
| Transplant programs | None | 868 (32.8) | 1,776 (67.2) | 0.001 |
|  | 1-2 in-house programs or 3 sharing programs | 97 (24.5) | 299 (75.5) |  |
|  | 3+ in-house programs | 24 (42.9) | 32 (57.1) |  |

^a^Data are shown as number (%) unless otherwise indicated.
